# Exposure to New Emerging Bisphenols Among Young Children in Switzerland

**DOI:** 10.3390/ijerph17134793

**Published:** 2020-07-03

**Authors:** Fiorella Lucarini, Tropoja Krasniqi, Gaëlle Bailat Rosset, Nicolas Roth, Nancy B Hopf, Marie-Christine Broillet, Davide Staedler

**Affiliations:** 1 Department of Biomedical Sciences, University of Lausanne, 1011 Lausanne, Switzerland; fiorella.lucarini@unil.ch (F.L.); tropoja.krasniqi@unil.ch (T.K.); marie-christine.broillet@unil.ch (M.-C.B.); 2 Scitec Research SA, Av. De Provence 18, 1007 Lausanne, Switzerland; gbailat@scitec-research.com; 3 Swiss Centre for Applied Human Toxicology (SCAHT), University of Basel, 4055 Basel, Switzerland; nicolas.roth@unibas.ch (N.R.); nancy.hopf@unisante.ch (N.B.H.); 4 Center for Primary Care and Public Health (Unisanté), University of Lausanne, 1007 Lausanne, Switzerland

**Keywords:** bisphenol analogues, biomonitoring, endocrine disruptors, urine, children’s health

## Abstract

Restrictions on the use of bisphenol A (BPA) in consumer products led to its replacement by various bisphenol (BP) analogues, yet young children’s exposure to these analogues has been poorly characterized so far. This study aimed to characterize infants’ and toddlers’ exposure to BPA and 14 emerging BP analogues (i.e., bisphenol AF, bisphenol AP, bisphenol B, bisphenol BP, bisphenol C (BPC), bisphenol E, bisphenol F (BPF), bisphenol G, bisphenol M (BPM), bisphenol P, bisphenol PH, bisphenol S (BPS), bisphenol TMC, and bisphenol Z). We extracted infants’ and toddlers’ urine from diapers (*n* = 109) collected in Swiss daycare centers as a practical and noninvasive alternative approach to urinary biomonitoring. Bisphenols were present in 47% of the samples, with BPC and BPM being the most frequently detected (23% and 25% of all samples, respectively). The mean concentrations of urinary BPS and BPF were greater than that of BPA. This contrasts with data reported previously. Furthermore, statistical analysis revealed a significant and negative correlation between urinary BPM concentration and the population’s age. Our results provide a first characterization of infants’ and toddlers’ exposure to bisphenols in Switzerland. This knowledge can be used to support ongoing biomonitoring studies and to prioritize exposure reduction and prevention strategies.

## 1. Introduction

Bisphenols (BPs) are a wide group of chemicals extensively utilized in polycarbonate plastics and epoxy resins, especially for the production of plastic packages for foods and beverages, dental sealants, thermal papers, medical devices, and toys. Among BP analogues, bisphenol A (BPA) is the most commonly used, with an annual production that reaches over 7.7 million tons, and is present in several consumer products [1]. Biomonitoring measurements of BPA in human serum, urine, hair, and breast milk have revealed widespread low-dose exposure to BPA in the EU and U.S. population [2,3,4,5,6,7,8]. A large body of experimental and epidemiological literature is available that links BPA exposure to a wide range of adverse health outcomes, and the evidence has been (or is currently being) reviewed by several governmental organizations [9,10,11,12,13,14,15,16].

BPA is a presumed human reproductive toxicant and is classified as Repro. Cat 1B, H360f [*May damage the fertility or the unborn child*] under EU Classification, Labelling and Packaging (CLP) Regulation (EC/1272/2008) (EC 2008). 

In 2017, the European Chemical Agency (ECHA) added BPA to the Candidate List of Substances of Very High Concern (SVHC), based on evidence for endocrine-disruption-mediated effects on various reproductive, cognitive, and metabolic endpoints [17]. There is some concern that fetuses, infants, toddlers, and children may be particularly vulnerable to adverse effects of BPA due to the vulnerability of developing organs to endocrine disrupting chemicals, particularly the brain, following pre- and/or postnatal exposure to BPA [16]. Indeed, early exposure to BPA has been associated with an increased risk of altered cognitive function (e.g., learning, memory) and behavior (e.g., hyperactivity), metabolic disorders (e.g., obesity), and breast or prostate cancer developed in later life [18,19,20,21]. In order to reduce the exposure to BPA in young children, BPA was banned in baby bottles in Canada, Europe, and USA [15,22]. Restrictions on the use of BPA in consumer products have led to its replacement by a number of BP analogues. Recently, more than 200 BPs have been identified as BPA analogues in consumer goods [23,24,25]. However, only a few have been considered relevant to human exposure even though they have widespread use (Table 1) and their presence has been documented in the environment.

Our work focused on the most significant BPA analogues selected on the basis of previous studies and includes bisphenol AF (BPAF), bisphenol AP (BPAP), bisphenol B (BPB), bisphenol BP (BPBP), bisphenol C (BPC), bisphenol E (BPE), bisphenol F (BPF), bisphenol G (BPG), bisphenol M (BPM), bisphenol P (BPP), bisphenol PH (BPPH), bisphenol S (BPS), bisphenol TMC (BPTMC), and bisphenol Z (BPZ) [26,28] (Table 1). The selected compounds are structurally similar to BPA with analogous physicochemical properties (Table 1), but their effects on animals and humans have been poorly studied [30,31]. Little is known about the toxicity of alternative BPs, and most of the toxicological information is limited to their endocrine disrupting potential [23]. Some studies suggested that these BPs have biological activities comparable to those of BPA due to their structural similarity [32,33,34]; other studies suggested that they have endocrine and toxicological activities even greater than BPA [35,36,37]. A systematic review by Rochester et al. showed that BPF and BPS exhibit hormonal activity with similar estrogenic, androgenic, and antiestrogenic potencies across different in vitro assays, and of the same order of magnitude as BPA [38]. Other studies have shown that BPB, BPC, and BPE exhibit estrogenic and antiandrogenic activities [24,32,39,40,41]. In vitro studies on zebrafish showed that BPS exposure (>0.5 μg/L) led to reproductive disruption decreasing egg production and gonadosomatic index [30,31]. Furthermore, it was observed that BPAF, BPB, and BPC exhibit estrogenic potencies similar to or greater than that of BPA. Antiandrogenic activity has been reported for BPAF, BPE, BPF, and BPB [28]. Moreover, in vitro and in vivo studies suggested other effects mediated by BPs, such as genotoxicity, oxidative stress, serum protein binding, and metabolic disorders [23,35,36,42,43,44].

Previous works suggested that infants and toddlers may have substantial exposure to BPs because of frequent contact with plastic objects such as baby bottles, teething rings, and toys, leading to hand-to-mouth transfers [45,46,47]. Available data on exposure to BP analogues, however, are very limited, and only a few studies have focused on the detection of urinary levels of these bisphenols in infants and toddlers [8,28,38]. Indeed, only nine (i.e., BPA, BPAF, BPAP, BPB, BPF, BPM, BPP, BPS, and BPZ) of the most common BPs have previously been analyzed in urine obtained from adults and children [8,28,48,49,50,51,52,53]. The analysis of human urine is a preferred approach in monitoring total BP concentrations, as urinary excretion is the main route of BP elimination, independently of the exposure route [49,51].

There is a need to characterize human exposure to BPs from various consumer products and to conduct more epidemiological research on the association between exposure to these emerging BPs and potential adverse health outcomes. In this regard, biomonitoring is a useful tool to quantify human exposure to BPs, and this approach can help in human health risk assessments [54].

As a first step in understanding BP exposure among children, we analyzed 15 bisphenols (bisphenol A, bisphenol AF, bisphenol AP, bisphenol B, bisphenol BP, bisphenol C, bisphenol E, bisphenol F, bisphenol G, bisphenol M, bisphenol P, bisphenol PH, bisphenol S, bisphenol TMC, and bisphenol Z) in urine extracted from diapers of infants and toddlers [55,56] attending Swiss daycare centers. Urine extracted from used diapers is a reliable sampling procedure, and sufficient quantities of urine can be obtained for the chemical analytical methods [57,58,59]. This sampling approach allowed us to investigate internal BP dose distributions in this young population at risk.

To the best of our knowledge, this is the first study focusing on the analysis of such a large number of BPA analogues in human urine; in addition, it also included BPPB, BPC, BPG, BPE, BPPH, and BPTMC, which have never before been assessed in infants’ and toddlers’ urine.

## 2. Materials and Methods

### 2.1. Chemicals

Bisphenol A (≥99%), bisphenol AF (≥99%), bisphenol AP (≥99%), bisphenol B (≥98%), bisphenol BP (≥98%), bisphenol C (≥98%), bisphenol E (≥98%), bisphenol F (≥98%), bisphenol G (≥98%), bisphenol M (≥99%), bisphenol P (≥99%), bisphenol PH (≥99%), bisphenol S (≥98%), bisphenol TMC (≥97%), and bisphenol Z (≥99%) were purchased from Sigma-Aldrich. Stock standard solutions (100 mg/L) were individually prepared by dissolving standard compounds in acetonitrile. A stock standard mixture containing all the individual standards (100 mg/L) was also prepared and stored at −20 °C. Working solutions for calibrations were prepared by serial dilution of the stock standard mixture with acetonitrile (Sigma-Aldrich). Beta-glucuronidase from *E. coli* (200 units/mL) was purchased from La Roche AG group. Bis(trimethylsilyl)trifluoroacetamide (BSTFA) with 1% of trimethylchlorosilane (TMCS) solution was used for BPs derivatization and was purchased from Sigma-Aldrich. Ammonium acetate (98%), calcium chloride (CaCl_2_) (≥99%), creatinine hydrochloride (≥97%), ammonium phosphate dibasic (≥99%), magnesium chloride hexahydrate (≥99%), potassium chloride (≥99%), sodium sulphate (≥99%), urea (≥98%), acetone (99.9%), formic acid (≥95%), and methanol (99.9%) were purchased from Sigma-Aldrich. Nanopure water was provided by an ultrapure water system (ariumPro, Sartorius, Germany).

### 2.2. Artificial Urine

A solution of artificial urine was prepared for calibration and blank controls to have a uniform matrix effect. The solution was adapted from Hu et al. [58] and from CSN EN 1616 (DIN EN 1616) and is composed of 1.2 g of creatinine hydrochloride, 3.4 g of ammonium phosphate dibasic, 0.5 g of magnesium chloride hexahydrate, 4.5 g of potassium chloride, 21 g of sodium sulphate, 1.5 g of urea, and 0.32 g of calcium chloride dehydrate. All compounds were dissolved in nanopure water, then stored at 4 °C [60].

### 2.3. Urine Sample Extraction

Urine samples were extracted from used disposable diapers (*n* = 109) with small modifications of a method reported by Hu et al. [58]. Briefly, the absorbent pad (i.e., polyacrylate) was removed from the diaper and placed into a flask containing 150 mL of CaCl_2_ at 150 g/L in nanopure water. The mixture was stirred 30 min in a shaker at 180 rpm at room temperature and filtered using a paper filter (Whatman, GE Healthcare Life Sciences; Chicago, Illinois, USA). The filtrate containing the expressed urine was placed in a graduate cylinder to quantify the total volume obtained, which was stored at −20 °C before analysis of BPs.

### 2.4. Chemical Analysis of BPs

The amount of BPs quantified in urine samples included unconjugated BPs and glucuronide conjugate metabolites. Glucuronidation of BPs in the intestine and liver is considered the main metabolic pathway for most bisphenols. These metabolites are mainly excreted through urine, and other metabolites only result when the glucuronidation pathway is saturated [41,57]. The total amount of free and glucuronide-conjugate BPs was extracted from samples by enzymatic deconjugation followed by Solid Phase Extraction (SPE) according to Hu et al. [58]. For each sample, the solution extracted from the diapers was buffered at pH 5.5–6 with ammonium acetate, then treated with 80 μL of β-glucuronidase to hydrolyze the glucuronide conjugates, and kept overnight at 37 °C [57,59]. BPs were extracted and purified using a SPE-offline protocol based on Chromabond Easy column (3 mL, 200 mg, Macherey-Nagel) [61]. Samples were loaded on a Solid Phase Extraction (SPE) cartridge preconditioned with 6 mL of methanol and 6 mL of water. The cartridge was washed with 3 mL of water (pH = 2 with HCl), and the compounds were eluted with 9 mL of acetone and formic acid (in ratio 99:1). After solvent evaporation, BPs were derivatized using BSTFA with 1% TMCS for 30 min at 75 °C and analyzed by gas chromatography-mass spectrometry (GC-MS, model GCMS-QP2010 Ultra, Shimadzu Corporation, Japan) equipped with an amine column (Rtx-5, length = 30 m, column diameter = 0.25 mm and thickness = 0.25 μm) and operated by the LabSolutions GC-MS Shimadzu software. Helium was used as carrier gas with a flow rate at initial temperature of 5 mL/min. The injection occurred at 280 °C in a splitless mode, the temperature ramp started at 45°C, increased to 300 °C at 10 °C/min, and then was kept at 300 °C for 5 min.

The ion source temperature was adjusted to 250 °C, and the mass spectrometer detector was set in the full scan acquisition mode to first identify the BPs. A selected ion monitoring (SIM) mode was then used to quantify the analytes in the samples and the standard mixture (Table 2). Creatinine analyses were performed by the company Unilabs Ticino (Breganzona, Switzerland), a certified bio-medical laboratory (ISO 15189 and ISO 17025), using the kinetic alkaline picrate method. BP urinary concentrations were adjusted to the concentration of creatinine and expressed in micrograms of BPs per gram of urinary creatinine (μg/g) and in micrograms per liter of urine (μg/L).

### 2.5. Calibration and Blank Assessment

Fifty mL of artificial urine were spiked with the standard mixture of BPA, BPAF, BPAP, BPB, BPBP, BPC, BPE, BPF, BPG, BPM, BPP, BPPH, BPS, BPTMC, and BPZ at 1, 2, 20, 60, 120, and 300 μg/L and added to unused disposable diapers. After incubation at room temperature (5 min), diapers were processed with the same procedure described for the analysis of the samples and used for calibration. Background analyte concentrations in disposable diapers were evaluated by pouring 50 mL of (i) nanopure water and (ii) artificial urine in unused disposable diapers. Different diaper brands (*n* = 23) among the most used in Switzerland were tested. The analysis of artificial urine extracted from diapers of different brands did not show the presence of the investigated BPs at concentrations higher than the limits of detection (LODs). The blanks were subjected to the same extraction protocol of the samples. Concentrations of the target analytes measured in blank controls were below the limits of detection. To assess the efficiency of the extraction from diapers, we calculated the recoveries of unconjugated BPA spiked in artificial urine at three concentrations: 1, 60, and 200 μg/L. Unused diapers were wetted with 50 mL of spiked artificial urine which was extracted as previously described. The recoveries of BPA were near quantitative (96%), and the same value was assumed for the other BPs. The limits of quantification (LOQs) and LODs (in parenthesis) were between 0.3–0.5 (0.09–0.15) μg/L.

### 2.6. Study Population

This study was based on infants and toddlers aged between 6 and 36 months, living in Switzerland. The mean age of the population was 20.2 months, and there were more boys than girls (63% vs. 37%). We obtained the samples (*n* = 109) from daycare centers, from 9 am to 12 o’clock, between June and July (2019). The samples were stored at −20 °C immediately after collection. This study was approved by the local ethics committee (CER-VD). Written consent was obtained from all parents/guardians of infants and toddlers that participated in this study before enrolment. To maintain anonymity, only age and sex of infants and toddlers were associated with the samples, and one diaper per child was collected to avoid unknown duplicates.

### 2.7. Statistical Analysis

**Figure 1:**
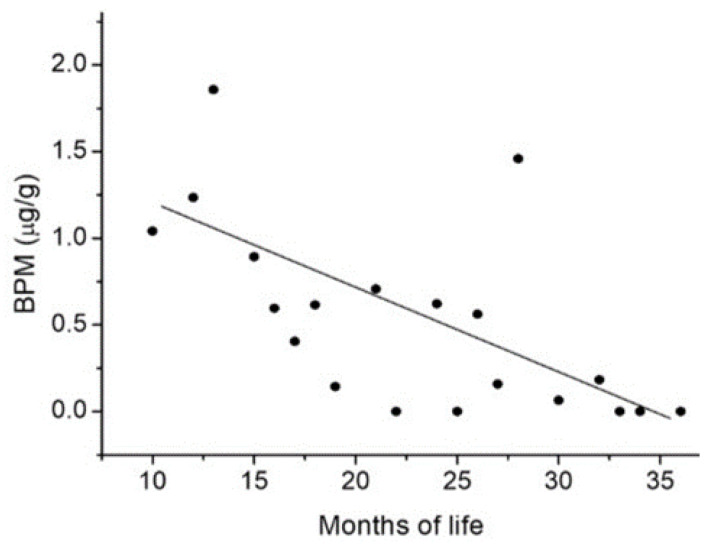
Graph showing the decline in average bisphenol M(BPM) concentrations (μg/g) (*y*-axis) by age (months of life) of infants and toddlers (*x*-axis).

Data were summarized using Microsoft Excel 2013 (Microsoft 365, Microsoft Corporation, USA), mean concentrations were calculated on volume-based and creatinine-adjusted basis for positive samples. Statistical analyses were performed using the R3.4.3 statistical package (R Foundation for Statistical Computing, Vienna, Austria). The influence of age and sex on the results obtained for each BP was analyzed using a generalized linear model (GLM) corrected for the binomial family based on the presence (≥LOQ = 1) and absence (<LOQ = 0) of BPs. The plot of BPM concentration (μg/g) vs. age (in months) of infants and toddlers (Figure 1) was obtained from the average concentrations of BPM per months of life. In the case of infants or toddlers with same months of life and detectable concentrations of BPM, an average concentration between the values was used on the *y*-axis.

## 3. Results

In our study, 47% of the samples were found to test positive for BPs, and 7 of the 15 BPs analyzed were detected. The concentrations of BPs correspond to the total amount of BPs in urine, resulting from unconjugated BPs and glucuronide-conjugate metabolites. Average mean concentrations and detection frequency for each BP were calculated and are reported in Table 3.

The highest detection frequency was observed for BPM and BPC, which were found in 25% and 23% of all samples, respectively. The BPs with the highest urinary concentrations were BPM (8.56 μg/g), BPP (1.85 μg/g), and BPC (1.03 μg/g); meanwhile, lower concentrations were detected for BPF (0.06 μg/g) and BPA (0.01 μg/g). Among the positive samples, 60% showed the presence of BPM. The concentrations of BPAF, BPB, BPG, BPZ, BPTMC, BPAP, BPBF, and BPPH were below the LOD. Correlations between the presence of BPs and the sex and age of the infants and toddlers were analyzed. We found that the presence of BPM significantly decreased with the age of infants and toddlers (*p*-value < 0.05) (Figure 1) but was not influenced by sex (*p*-value > 0.1). Other BPs tested were not significantly influenced by age or sex (*p*-value > 0.1).

## 4. Discussion

BPM and BPC were detected in about 1 in 4 samples, while BPAF, BPB, BPG, BPZ, BPTMC, BPAP, BPBF, and BPPH were not detected.

BPM is used as an ingredient to synthesize a blend of diphthalonitrile ether resins [62]. This BP has been rarely analyzed in human biomonitoring studies. Rocha et al. found no detectable concentrations of BPM in 300 urinary samples from Brazilian school children aged 6 to 14 years collected in 2012–2013 [50].

Data on urinary concentrations of BPC, BPPH, BPG, and BPE in human populations have never before been published. To date, the presence of these BPs have only been detected in human breast milk at concentrations lower than the limit of quantification [26,63]. Among the BPs analyzed, BPA, BPF, and BPS have been the most frequently detected and investigated in the literature [23]. In our study, BPA was detected in 7% of the samples, with a mean concentration of 2.4 μg/L (0.06 μg/g creatinine). BPA concentrations were similar to the concentration of BPF (2.7 μg/L, 0.07 μg/g, mean values), while a higher concentration was detected for BPS (6.41 μg/L, 0.15 μg/g, mean values). Compared to our results, BPA was more frequently detected (range from 74 to 100%) in urine samples of children collected from 2006 to 2014 in different countries such as USA, Greece, Germany, and Spain [8,64,65,66]. In the present study, we observed a decreased detection frequency of BPA with respect to other BPs, which can be explained on the basis of the restrictions on the use of BPA that several countries have adopted over the past few years.

Internal dose values for BPA were greater than for the other BPs. The BPA concentrations that we detected in this study were slightly higher than those found in urine samples collected in 2014 in the National Health And Nutrition Examination Survey (NHANES) study in the USA (1.3 μg/L, median value, *n* = 409) for children aged between 6 and 11 years [8]. In contrast, urinary BPA levels (2 μg/L, geometric mean (GM)) reported for Greek young children (aged 2–3 years) sampled in 2009–2011 were comparable to our BPA concentrations (2.4 μg/L) [64]. Others have reported urinary BPA concentrations of 4, 3.5, and 3.1 μg/L (as GM) obtained from toddlers (age <5 years) from China, Germany, and Spain, respectively. These were higher compared to our BPA values [57,65,66]. The similarities and differences observed might be due to geographical locations, age groups included in the study, and governing regulations. Nevertheless, the current tolerable daily intake (TDI) determined by the European Food Safety Authority for BPA in 2015 is 4 μg/kg-day, which is lower than the estimated median BPA exposures calculated from international studies (0.02–0.12 μg/kg-day for children) [67]. TDIs for the other BPs do not yet exist.

Only limited data regarding the detection and quantification of BPF and BPS are available in both adult and children exposure studies. Reliable chemical analytical methods including LODs and LOQs need to be established to be able to compare data across biomonitoring studies. A study conducted with USA children (aged 6–11 years) in 2014 reported lower median levels of BPF (0.27 μg/L) and BPS (0.27 μg/L) compared to our findings and a lower detection frequency with respect to BPA [8]. Other BPs have been sporadically detected in environmental and human samples [26,28]. In our study, the urinary BPAF concentrations were lower than the LOD. Other studies have reported detectable BPAF concentrations [68,69,70,71], with the highest concentrations (0.018 μg/L) reported in a Chinese cohort in 2013 [72].

Exposure data for BPs other than BPA are scarce. The findings of our study showed that average BPM concentrations are higher in infants than in toddlers. We can only hypothesize that these results are related to different sources of exposure for infants and toddlers. Infants may ingest contaminated food and beverage, while toddlers could potentially be exposed from eating dust (hand to mouth) generated from the building materials, as BPM has been reported used in coating, adhesives, electronics, and structural applications as well as in automobiles. Urinary presence of BPM could also be an indicator of the presence of this BP in childcare products leading to skin absorption. However, it is difficult to identify the contribution of various exposure routes and sources to the overall internal dose, as no comprehensive BPM exposure characterization has been conducted among the investigated population. The advantage of biomonitoring is that it incorporates all routes of exposure and provides a total internal dose. The limitation of the biomonitoring approach is that the source of this body-burden cannot be assessed.

There is a need to characterize the various exposure sources of BP analogues in consumer and childcare products as well as in the indoor environment. Furthermore, as a consequence of restrictions on the use of BPA in consumer products, urinary BPA levels have decreased steadily over time in USA and EU populations [73], while urinary BP analogues levels are now on the rise. Despite the extensive amount of experimental data generated for BPA, our ability to link chronic low-dose environmental exposure to BPA with adverse human health effects remains so far limited. Controversies over low-dose effects of BPA exist as well as knowledge gaps and shortcomings in BPA basic biology data and dose-response analysis, and in the integration of academic research findings into regulatory risk assessment [74]. This situation has prompted several consortium-based initiatives to address the remaining areas of uncertainty [75,76,77]. Closing all the gaps will require massive research efforts.

Adverse outcome pathways (AOP)-based thinking has been proposed as a driver for generating new experimental data for BPA [74]. AOPs have been proposed recently for BPS [78] and BPF [79]. Similarly, applying exposure-based, pathway-oriented approaches (e.g., aggregate exposure pathways, AEPs) [80,81] can support a better characterization of the source-to-outcome continuum by exploring the linkages between exposure sources and pathways with internal exposure information (i.e., biomonitoring data), which can be then linked to AOPs. The value of integrating biomonitoring data to better support human health risk assessment and regulatory decision-making regarding chemicals has been widely acknowledged, yet the use of biomonitoring data and the level of implementation across sectorial chemical regulatory frameworks and regions vary [6,82]. Knowledge assembly tools like AOPs and AEPs can facilitate this integration and help focus and prioritize experimental and epidemiological research efforts on BPs to meet critical regulatory risk assessment needs.

This study represents a first snapshot of young children’s exposure to BP analogues; however, it has a number of limitations, both from an epidemiological and a methodological point of view. The number of samples investigated is too limited to provide results representative of a whole population. The extraction efficiency was investigated only for BPA, assuming the same behavior for the other BPs. A 24-h urine collection was not possible because of the setup of the study (i.e., protocol for sample anonymization). Despite these limitations, our results provide a first landmark characterization of infants’ and toddlers’ exposure to BPs in Switzerland, even though the single spot urine samples utilized in the study mainly reflect the exposure occurring shortly before urine collection [83]. However, further studies on a larger population scale could enable the single spot approach to accurately reflect the average exposure to BPs [83]. Furthermore, our study highlights the feasibility of using commercial diapers to monitor infants’ and toddlers’ exposure to new emerging bisphenols. In particular, these preliminary results can inform the ongoing debate of whether to establish a national program to monitor the environmental chemical exposure of the Swiss population [84,85].

## 5. Conclusions

Results from our study show that toddlers and infants are extensively exposed to BP derivatives; however, in comparison with the existing literature, the exposure to this class of compounds has changed over time, as demonstrated by the decreased BPA detection frequency and the increasing concentrations of other BPs. In this context, the mean urinary levels of BPS and BPF were higher than those measured for BPA, which is in contrast with data reported in the literature until now [8,28]. Information on emerging BPs is still scarce, and it is warranted to include these compounds in extended prospective studies with greater samples sizes to prevent the potential harmful human exposure to BPs and health consequences. Finally, these studies should be coupled with the analysis of BPs in products and cosmetics for childcare to assess BPM and BPC presence and migration from such products.

## Figures and Tables

**Table 1:** Structures and physicochemical properties of the bisphenols (BPs) investigated.

BP CAS n°	Structural Formula	Log k_ow_	Applications	Potential Exposure Sources	Ref.
**BISPHENOL A** (BPA) 80-05-7	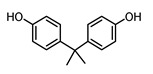	3.43	Polycarbonate plastics, epoxy resins	Thermal papers, food, beverages, dust, personal care products, textile products	[26] [23] [9] [5]
**BISPHENOL AF** (BPAF) 1478-61-1	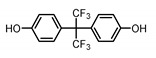	4.47	Coating products, epoxy resins	Food, beverages, dust, dental materials, personal care products, textile products	[23] [26] [9]
**BISPHENOL AP** (BPAP) 1571-75-1	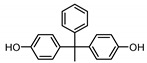	4.86	Coating products, epoxy resins	Food, beverages, thermal papers, dust, textile products	[23] [26] [9]
**BISPHENOL B** (BPB) 77-40-7	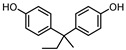	4.13	Coating products, epoxy resins	Food, beverages, dust, textile products	[23] [26] [27]
**BISPHENOL BP** (BPBP) 1844-01-5	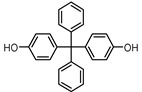	6.08	Flame retardants, polycarbonate plastics	Plastic products, furniture	[26]
**BISPHENOL C** (BPC) 14868-03-2	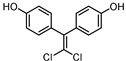	3.74	Flame retardants, polycarbonate plastics	Thermal papers, textile products	[26] [23]
**BISPHENOL E** (BPE) 2081-08-5	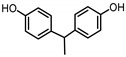	3.19	Coating products, polycarbonate plastics	Food, beverages, textile products	[26] [23]
**BISPHENOL F** (BPF) 620-92-8	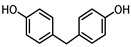	3.06	Coating products, lacquers	Food, adhesives, varnishes, textile products, dust	[23] [26] [27]
**BISPHENOL G** (BPG) 127-54-8	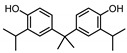	6.55	Polycarbonate plastics, epoxy resins	Dental materials	[26]
**BISPHENOL M** (BPM) 13595-25-0	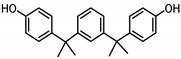	6.25	Thermoplastics, polycarbonate plastics	Plastic products, dental materials	[28]
**BISPHENOL P** (BPP) 2167-51-3	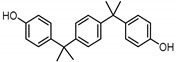	6.25	Polycarbonate plastics, epoxy resins	Food, dust	[23]
**BISPHENOL PH** (BPPH) 24038-68-4	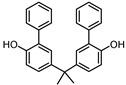	7.17	Polycarbonate plastics, epoxy resins	Food, beverages	[28]
**BISPHENOL S** (BPS) 80-09-1	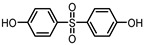	1.65	Coating products, dyes, leather tanning agents	Food, beverages, thermal papers, dust, textile products	[23] [26] [27]
**BISPHENOL TMC** (BPTMC) 129188-99-4	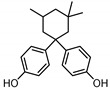	6.29	Polycarbonate plastics, epoxyr esins, polyesters	Dental materials, dust	[29] [28]
**BISPHENOL Z** (BPZ) 843-55-0	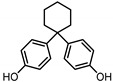	5.00	Coating products, polycarbonate plastics	Food, personal care products, textile products	[23] [26]

Log K_ow_ = octanol-water partition coefficient [28].

**Table 2:** List of target analytes with gas chromatography-mass spectrometry (GC-MS) parameters used for identification.

Derivatized Bisphenols	Retention Time (min)	Monitored Ions (*m*/*z*)
BPAF	18.85	480, 411, 225
BPF	20.26	344, 179, 157
BPE	20.52	358, 372
BPA	20.84	372, 357, 207
BPB	21.53	371, 357, 191
BPG	21.78	456, 441, 249
BPC	22.95	424, 354
BPZ	23.93	412, 369
BTMC	24.20	454, 397, 383
BPS	24.55	394, 379, 229
BPAP	24.83	434, 419, 269
BPM	26.12	490, 475, 207
BPP	27.40	490, 475, 207
BPBF	28.23	496, 419, 331
BPPH	28.40	509, 267, 247

**Table 3:** Summary of BPs detection frequency (%), mean concentrations (average calculated for positive samples), and concentration ranges (micrograms bisphenol per gram of urinary creatinine (μg/g) and μg/L in parenthesis).

Bisphenols Derivatized	Detection Frequency % (Case Numbers)	Mean μg/g Creatinine (μg/L)	Range (μg/g Creatinine (μg/L)
BPF	2 (2)	0.07 (2.7)	0.06–0.08 (1.80–3.61)
BPE	2 (2)	0.12 (5.85)	0.07–0.17 (2.7–9.00)
BPA	7 (8)	0.06 (2.40)	0.01–0.11 (1.22–3.30)
BPC	23 (25)	0.25 (11.22)	0.03–1.03 (1.84–52.34)
BPS	3 (3)	0.15 (6.41)	0.11–0.19 (2.60–12.02)
BPM	25 (27)	1.31 (53.07)	0.13–8.56 (6.18–273.48)
BPP	4 (4)	0.58 (20.54)	0.04–1.85 (4.38–49.36)
BPAF	<LOD	n.a.	n.a.
BPB	<LOD	n.a.	n.a.
BPG	<LOD	n.a.	n.a.
BPZ	<LOD	n.a.	n.a.
BPTMC	<LOD	n.a.	n.a.
BPAP	<LOD	n.a.	n.a.
BPBF	<LOD	n.a.	n.a.
BPPH	<LOD	n.a.	n.a.

n.a. = not available.
